# Hepatitis B vaccination and serology among health personnel in a municipality in the Recôncavo Baiano, Brazil, 2019

**DOI:** 10.47626/1679-4435-2022-975

**Published:** 2024-02-16

**Authors:** Yvanilson Costas Farias Junior, Fernanda de Oliveira Souza, Margarete Costa Heliotério, Tânia Maria de Araújo, Paloma de Sousa Pinho

**Affiliations:** 1 Núcleo de Saúde, Educação e Trabalho, Universidade Federal do Recôncavo da Bahia, Santo Antônio de Jesus, BA, Brazil; 2 Núcleo de Epidemiologia, Universidade Estadual de Feira de Santana, Feira de Santana, BA, Brazil

**Keywords:** vaccination, hepatitis B, health personnel, serology, vacinação, hepatite B, pessoal de saúde, sorologia

## Abstract

**Introduction:**

Health personnel are more susceptible to contamination by the hepatitis B virus due to occupational risk and need special care. Previous studies have found, however, that not all health personnel were fully vaccinated against hepatitis B, as recommended by the Ministry of Health.

**Objectives:**

To analyze the factors associated with full hepatitis B vaccination and to evaluate post-vaccination serological response among health personnel.

**Methods:**

Cross-sectional study, conducted in the municipality of Santo Antônio de Jesus, Bahia, Brazil. The sample consisted of 453 health personnel from primary and medium-complex care.

**Results:**

The prevalence of full hepatitis B vaccination among health personnel was 56.9%. The variables associated with the prevalence of complete hepatitis B vaccination in the final analysis model were: working in primary health care (prevalence ratio = 1.31; 95% CI 1.04-1.65) and medicine preparation or administration (prevalence ratio = 3.53; 95% CI 2.17-5.74). Around 72% of those who reported being shot with all three doses of the hepatitis B vaccine had been tested for circulating antibodies in their blood and 88.4% were immune to the hepatitis B virus.

**Conclusions:**

The familiarity provided by routine primary health care and the awareness of occupational risk was associated with beter adherence to the hepatitis B vaccine schedule among health personnel. Nearly a third of those who were shot with the three doses of hepatitis B vaccine were not immunized, reinforcing the need for anti-HBs testing.

## Abstract (pt)

**Introdução:**

Devido à maior susceptibilidade à contaminação pelo vírus da hepatite B em virtude do risco ocupacional, trabalhadores(as) da saúde necessitam de cuidado especial. Entretanto, estudos prévios constataram que nem todos os profissionais apresentavam esquema vacinal completo para hepatite B, como é preconizado pelo Ministério da Saúde.

**Objetivos:**

Analisar os fatores associados à vacinação completa para hepatite B e avaliar resposta sorológica pós-vacinação entre trabalhadores(as) da saúde.

**Métodos:**

Estudo transversal, realizado no município baiano de Santo Antônio de Jesus, cuja amostra foi composta por 453 trabalhadores(as) da saúde da Atenção Primária à Saúde e da média complexidade.

**Resultados:**

A prevalência de vacinação completa para hepatite B entre trabalhadores(as) da saúde foi de 56,9%. No modelo final de análise, as variáveis associadas à prevalência de vacinação completa para hepatite B foram: trabalhar no nível da Atenção Primária à Saúde (razão de prevalência = 1,31; IC95% 1,04-1,65) e preparo ou administração de medicamentos (razão de prevalência = 3,53; IC95% 2,17-5,74). Daqueles(as) que relataram o recebimento das três doses da vacina para hepatite B, 88,4% realizaram a testagem de anticorpos circulantes no sangue, e cerca de 72% estavam imunes ao vírus da hepatite B.

**Conclusões:**

O conhecimento proporcionado pela rotina na Atenção Primária à Saúde e o reconhecimento do risco ocupacional estava associado à melhor adesão à vacina para hepatite B entre trabalhadores(as) da saúde. Quase um terço dos(as) trabalhadores(as) que receberam as três doses da vacina para hepatite B não estava imunizado, reforçando a necessidade da realização do exame anti-HBs.

## INTRODUCTION

It is estimated that 257 million people (3.5%) are living with chronic hepatitis B infection worldwide.^1,2^ In Brazil, 689,933 confirmed cases of viral hepatitis were reported in the Notifiable Diseases Information System (Sistema de Informação de Agravos de Notificação, SINAN) from 1999 to 2020, and 38.1% were hepatitis B infections. According to the Mortality Information System (Sistema de Informação sobre Mortalidade, SIM), 78,642 deaths related to viral hepatitis were recorded between 2000 and 2019, and 21.3% were associated with type B viral hepatitis. Hepatitis B infection rates have shown a slight downward trend over the last 5 years.^3^

Vaccination against hepatitis B began for groups at high risk of hepatitis B virus infection throughout Brazil in 1998. The World Health Organization (WHO) recommends universal immunization, regardless of prevalence levels, including it in the routine immunization schedule for children shortly after birth. In Brazil, the hepatitis B vaccine was introduced into the vaccination schedules for children, adolescents, adults, and older people in 2004.^4^ Currently, the hepatitis B vaccine is administered in a three-dose schedule, with a 1-month interval between the first and second doses and a 6-month interval between the first and third doses.^2,5^

Hepatitis B virus transmission occurs through parenteral, sexual, and vertical transmission. These groups are considered to be at risk of infection: Newborns of offspring carrying the surface antigen of the virus (HBsAg), drug users, dialysis patients, and health personnel.^2,5^

In view of the highly infectious nature of the hepatitis B virus and the potential for transmission in different ways, such as accidents involving exposure to biological materials and sharps accidents, as a result of the occupational risk to which health personnel are exposed, they require special care, as they are more susceptible to hepatitis B virus contamination when compared to the general population.^6,7,8^ In this context, the importance of vaccination against hepatitis B is evident, as it has been proven to be the most effective and safest prophylactic measure to combat the hepatitis virus and its occupational transmission in health care setings.^2^

Despite the undeniable importance of full hepatitis B vaccination, in a systematic review of African countries, hepatitis B vaccination coverage among health personnel was 24.7%. The highest coverage was found in North Africa (62.1%), and the lowest in Central Africa (13.4%).^9^ Studies conducted in Sudan also found sub-optimal vaccination among hospital personnel, 41 and 72.6%, respectively.^10,11^

In Brazil, studies with primary health care (PHC) and medium-complexity health personnel in different cities across the country found that not all health personnel had a full vaccination schedule for hepatitis B, as is recommended by the Ministry of Health (MoH).^6,7,8,12^ Furthermore, among health personnel with a full vaccination schedule for hepatitis B, not all of them had been screened for seroconverted through an anti-HBs test, which MoH recommends.^6,7,8,12,13,14,14^

Surveys investigating hepatitis B vaccination among health personnel outside hospital settings are still incipient. Studies on hepatitis B vaccination among medium-complexity health personnel are scarce.^7,8^

In this scenario, this study involves essential issues concerning the health of PHC and medium-complexity health personnel, who are directly in contact with patients who may be exposed to various vaccine-preventable diseases. Therefore, this study aims to analyze factors associated with full hepatitis B vaccination and to evaluate post-vaccination serological response among PHC and medium-complexity health personnel.

## METHODS

This is a cross-sectional study conducted in the municipality of Santo Antônio de Jesus, BA, Brazil, in 2019, as part of the multicenter project *Vigilância e monitoramento de doenças infecciosas entre trabalhadores e trabalhadoras do setor saúde*. The study population consisted of 453 PHC and medium-complexity health personnel, both directly related to care and administrative, general service, security, and other tasks.

The sample was calculated considering the total population of health personnel (622), a prevalence of full hepatitis B vaccination of 79.2%,^7^ an error of 3%, and a 95% confidence level. A representative sample of 332 health personnel was estimated, stratified by occupational group and level of care to investigate the outcome of interest. As this was a broader study which investigated different health outcomes, the sample size was larger than that estimated for the analysis of vaccination status.

The study population was randomly selected by stratified sampling, defined by means of a previous survey of the structure of the municipality’s network and workforce, considering the complexity level of the services and occupational groups. The selection involved a list of all health personnel in the services included in this study. A random number list was drawn using the Statistical Package for Social Sciences (SPSS), version 22.0 (IBM Corp, NY, USA), considering the strata mentioned (level of care: PHC and medium complexity), and occupational groups.

Data were collected between May and December 2019, using a structured instrument containing nine blocks of questions, including questions on social conditions, health, exposure at work, and vaccination. Upon arriving at the workplace of the selected health personnel, the interviewers briefly explained the methods, aims, and objectives of the research. After the participant had answered the questionnaire, the examination team scheduled the test collection.

The interviewers were calibrated through training to check for possible inconsistencies in the survey instrument and to look for strategies to optimize the interview time. The anti-HBs test was performed by a laboratory associated with the study. Anti-HBs values > 10 IU/mL were used as the cut-off point for determining adequate levels of protective antibodies.

The outcome variable was a hepatitis B vaccination report. The analysis of this outcome included an assessment of full hepatitis B vaccination considering the adult vaccination schedule recommended by the National Immunization Program (*Programa Nacional de Imunização*, PNI). The prevalence of hepatitis B vaccination was determined based on these questions: Have you ever been vaccinated against hepatitis B? Yes; No; Don’t know/don’t remember. If yes, did you receive: 1 dose; 2 doses; 3 doses; don’t know.

The independent variables were grouped as follows: sociodemographic characteristics (sex, age, children, schooling, marital status, and skin color); occupational characteristics (level of health care and employment relationship); occupational exposure (contact with biological material, chances of injury, and medication preparation or administration).

Training staff to enter the data was based on how to use the statistical software Statistical Package for the Social Sciences (SPSS), version 23.0, for Windows. The data were double-entered, and the questionnaires were exchanged among the operators.

Statistical analyses were based on SPSS and STATA (Software for Statistics and Data Science). Initially, a descriptive univariate analysis was performed using SPSS.

Bivariate analysis was conducted to test the association between the dependent variable (full hepatitis B vaccination) and the categorical exposure variables. Prevalence ratios (PR) and their respective 95% confidence intervals (95% CI) were calculated. Pearson’s chi-squared test was used to assess the measure of statistical significance, using p-values < 0.05.

Finally, a multivariate analysis was performed to describe the simultaneous effect of the variables of interest on the completion of the vaccination schedule. The variables were selected on the basis of a literature review, model assumptions were checked, and the variables were shortlisted, considering a p-value ≤ 0.20 in the bivariate analysis. The logistic regression model with correction of the odds ratio (OR) for PR was performed using Poisson regression with robust variance, and their respective CI.^15^

The Research Ethics Committee of the State University of Feira de Santana approved this study CAAE 90204318.2.0000.0053, and it followed the recommendations of Resolution 466/12 of the Brazilian National Health Council.

## RESULTS

A total of 453 health personnel from the municipality of Santo Antônio de Jesus, BA, Brazil, participated in the study, 352 from PHC and 101 from medium-complexity care. The predominant proportion of the health personnel studied were women (82.8%) and Black (83.6%); 76% were aged between 21 and 49; 73.9% had children; 60.9% reported being partnered, and 38.2% had completed higher education.

As for working conditions, the majority worked in PHC (77.7%) and reported having a permanent job (72.4%). As for exposure at work, 55.1% reported coming into contact with biological materials; 49.2% were likely to be injured, and 24.3% prepared or administered medicines (Table 1).

**Table 1:** Prevalence of full hepatitis B vaccination (three doses) among Primary Health Care and medium-complexity health personnel, according to sociodemographic, job, and occupational exposure, Santo Antônio de Jesus, BA, Brazil, 2019

Variables	n (%)	Prevalence of full vaccination n (%)	PR	95%CI
Sex (n = 453)				
Female	375 (82.8)	219 (58.4)	1.32	0.88-1.97
Male	78 (17.2)	39 (50.0)	-	-
Age (years) (n = 442)				
21-49	336 (76.0)	188 (56.0)	0.84	0.59-1.18
50+	106 (24.0)	65 (61.3)	-	-
Children (n = 444)				
Yes	328 (73.9)	185 (56.4)	0.91	0.66-1.25
No	116 (26.1)	69 (59.5)	-	-
Marital status (n = 447)				
Partnered	272 (60.9)	168 (58.8)	1.13	0.90-1.42
Not partnered	175 (39.1)	94 (53.7)	-	-
Education (n = 440)				
Higher education and beyond	168 (38.2)	101 (60.1)	1.06	0.91-1.23
Technical education and some higher education	272 (61.8)	153 (56.3)	-	-
Skin color* (n = 440)				
Other than Black	72 (16.4)	40 (55.6)	0.99	0.91-1.07
Black	368 (83.6)	209 (56.8)	-	-
Level of health care (n = 453)				
PHC	352 (77.7)	210 (59.7)	1.46	1.03-2.05
Medium complexity	101 (22.3)	48 (47.5)	-	-
Employment relationship (n = 442)				
Permanent	320 (72.4)	176 (55.0)	0.80	0.58-1.09
Temporary	122 (27.6)	76 (62.3)	-	-
Contact with biological material (n = 448)				
Yes	247 (55.1)	162 (65.6)	1.53	1.25-1.88
No	201 (44.9)	93 (46.3)	-	-
Chances of injury (n = 435)				
Yes	214 (49.2)	131 (61.2)	1.17	0.98-1.41
No	221 (50.8)	117 (52.9)	-	-
Medication preparation or administration (n = 448)				
Yes	109 (24.3)	91 (83.5)	1.40	1.27-1.56
No	339 (75.7)	164 (48.4)	-	-

*Other than Black (Yellow, White, and Indigenous) and Black (Brown and Black).

The 95%CI values in bold highlight the confidence intervals that did not include the value of 1.

PHC = Primary Health Care; PR = prevalence ratio.

The factors associated with reporting full hepatitis B vaccination among PHC and medium-complexity health personnel in the bivariate analysis (95% CI) included: working in PHC, having contact with biological materials, and medicine preparation or administration (Table 1).

The variables associated with prevalence of full hepatitis B vaccination in the final analysis model were: working in PHC (PR = 1.31; 95% CI 1.04-1.65) and medication preparation or administration (PR = 3.53; 95% CI 2.17-5.74) (Table 2).

**Table 2:** Variables found in the final regression model associated with the prevalence of full hepatitis B vaccination (three doses) among Primary Health Care and medium-complexity health personnel in Santo Antônio de Jesus, BA, Brazil, 2019

Variables (exposed)	Adjusted PR	95% CI	p-value
Level of health care (PHC)	1.31	1.04-1.65	0.018
Medication preparation or administration (yes)	3.53	2.17-5.74	< 0.001

PHC = Primary Health Care; PR = prevalence ratio. 95% CI = 95% confidence interval

**Figure 1:**
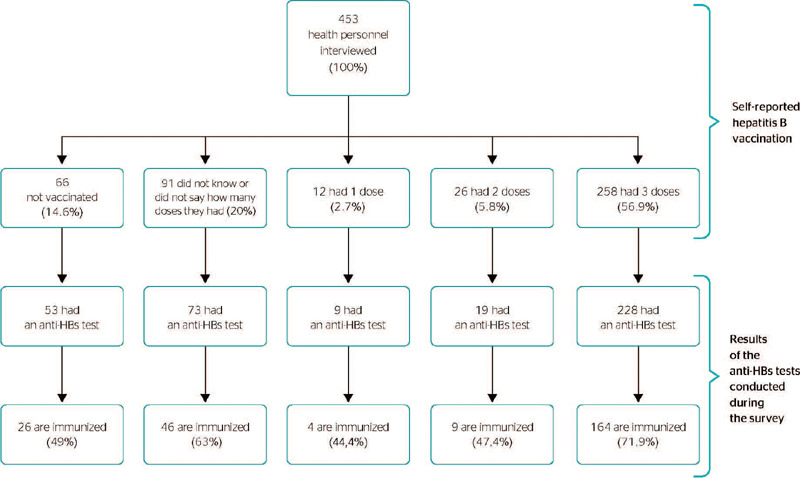
Flowchart showing the prevalence of full hepatitis B vaccination and serological testing (anti-HBs test) to determine immunity among Primary Health Care (PHC) and medium-complexity health personnel (n = 453), in Santo Antônio de Jesus, Bahia, Brazil, 2019.

When health personnel were asked about the completion of their hepatitis B vaccination schedule, only 56.9% reported full vaccination. As for serological tests to prove immunity, 88.4% of health personnel who reported receiving all three doses of the vaccine had been tested for circulating antibodies in their blood, and around 72% were immune to HBV. Therefore, no seroconversion occurred for approximately 28% of health personnel (Figure 1).

*Anti-HBs tests were not conducted on all 453 health personnel in the study due to study budget.

When analyzing hepatitis B seroconversion rates according to the number of doses reported by health personnel, less than 50% of those who received 1 or 2 doses became immune to the hepatitis B virus, whereas 71.9% of those who reported receiving three doses became immune (Figure 1).

**Figure 2:**
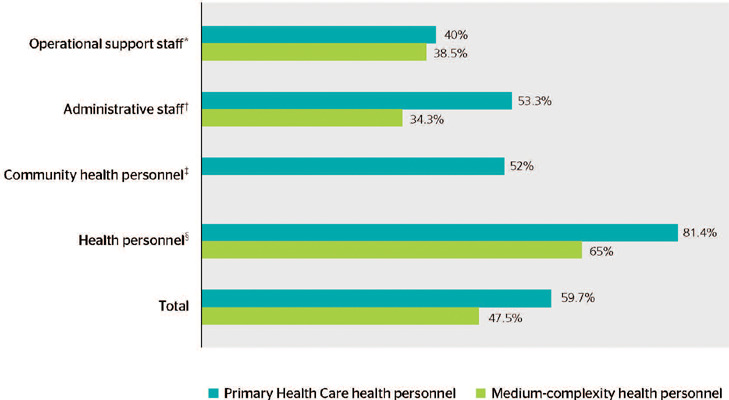
Prevalence of full hepatitis B vaccination among Primary Health Care (PHC) health personnel (n = 352) and medium-complexity health personnel (n = 101), according to occupation, in Santo Antônio de Jesus, BA, Brazil, 2019.

In total, 59.7% of PHC and 47.5% of medium-complexity health personnel reported a full hepatitis B vaccination schedule. The prevalence of full hepatitis B vaccination schedules was categorized according to occupation, showing a higher prevalence of full hepatitis B vaccination among health personnel in PHC (81.4%) and medium-complexity care (65%). On the other hand, operational support staff had the lowest full hepatitis B vaccination rates in PHC (40%), whereas administrative staff had the lowest (34.3%) in medium-complexity PHC (Figure 2).

*General helpers, security, drivers, etc.; ^†^ Administrative staf, coordinators, supervisors, etc.; ^‡^ Community health personnel and endemic disease control health personnel (work only in PHC); ^§^ Nurses, physicians, dentists, health personnel, etc.

## DISCUSSION

In this study, 56.9% of health personnel were fully vaccinated against hepatitis B. The level of health care, contact with biological material, and medicine preparation or administration were variables associated with full hepatitis B vaccination. Almost a third of the health personnel with a complete vaccination schedule did not have adequate titers of protective antibodies as screened by the anti-HBs test.

In this study, the prevalence was lower than that found in the same municipality, when a prevalence of 59.9% was found for full hepatitis B vaccination among PHC and medium-complexity health personnel.^7^ In an study involving nursing professionals in a public hospital in Paraíba, Brazil, 65.7% reported that they were fully vaccinated against hepatitis B.^14^ In Feira de Santana, Bahia, Brazil, the prevalence of full hepatitis B vaccination was 69.8%.^13^ Tus, hepatitis B vaccination coverage is less than ideal, not only in this study but also in other Brazilian municipalities.

This shows that although the completeness of hepatitis B vaccination is similar in the country, the picture is different abroad. A study with hospital health personnel in Austria found a 93.8% vaccination rate for hepatitis B.^16^ In France, hepatitis B vaccination coverage reached 88.2% among health students.^17^ A multicenter study conducted in 10 Italian municipalities found that 77.3% of health personnel were fully vaccinated against hepatitis B.^18^ In Catalonia, Spain, 75.6% (487) of the health personnel surveyed reported that they were fully vaccinated against hepatitis B, but only 39.8% (253) had proof of immunization on their vaccination records.^19^

A study at Osaka University Hospital found that 86.7% of the sample had been vaccinated with three doses of the hepatitis B vaccine.^20^ In Saudi Arabia, 83.5% of health personnel in government institutions were fully vaccinated against hepatitis.^21^ A survey with 120 health personnel in hospitals in China showed that only 60% had completed the hepatitis B vaccination schedule.^22^

These studies were conducted among health personnel with high risk of environmental exposure to biological agents, in which risk perception may be a factor that improves adherence to protective measures among these groups. On the other hand, the health system in Brazil, which is strongly based on PHC, favors health promotion and protection, and health personnel are closer to actions to encourage and foster vaccination, thus explaining the differences in the prevalence of full hepatitis B vaccination among PHC and medium-complexity health personnel.

However, on a national level, there has been a downward trend in vaccination coverage since 2015 for the various immunizers, including among age groups with a tradition of high coverage, such as children. Actions to encourage vaccination should be implemented at all levels of the health system. Longitudinal care, which involves monitoring individuals throughout their life cycle, should include vaccination. It is therefore essential that PHC, as a coordinator of care, can guarantee the continuity of actions and monitoring of vaccination among all age groups, which presupposes health personnel.

In this study, the highest prevalence of full hepatitis B vaccination was observed among PHC health personnel, and these individuals were 31% more likely to have completed the hepatitis B vaccination schedule than medium-complexity health personnel. This scenario is probably associated with vaccination being generally an integrated and routine action of health services at the PHC level, so teams from PHC and family health facilities play a key role in vaccine recommendations and administration.^5^

It is therefore assumed that the awareness gained through the routine of PHC services can offer health personnel a place where strategies to encourage vaccination are more frequent.^13^

Exposure to possible risks was also characterized as an element associated with a higher prevalence of full hepatitis B vaccination among health personnel, with contact with biological materials, and medication preparation or administration being associated with full hepatitis B vaccination. Health personnel with greater exposure were 3.5 times more likely to be fully vaccinated against hepatitis B. These findings may be related to the recognition of the occupational risk to which these health personnel are exposed, resulting in greater adherence to self-care measures, including hepatitis B vaccination.^7,8^

Health personnel, who provide direct patient care, had a higher prevalence of full hepatitis B vaccination. In previous studies, health personnel and nursing staff were the most likely to have been fully vaccinated against hepatitis B.^9,18,21,22^ In addition to the perception of occupational exposure, there is also a greater familiarity with the subject among these professionals, certainly due to having more years of schooling.

The perception of risk may be related to their familiarity with the subject, so the absence or lack of information is probably associated with low adherence to the vaccine. Different studies show that health personnel understanding of hepatitis B vaccination is incipient in various countries, including Brazil. Among the reasons cited as to why health personnel are not vaccinated are: belief in the ineffectiveness and unreliability in the vaccine, hepatitis B being an unusual and unlikely infection, difficulty in accessing the immunizer, and the cost of the vaccine.^6,9,21,22^

These factors, in addition to the health personnel unawareness of the need to assess their immunity, make them susceptible to infection. In addition to vaccination, it is recommended to confirm immunity through serology to detect the antibody against the hepatitis B surface antigen, known as the anti-HBs test.^7^ The PNI does not routinely recommend anti-HBs testing after hepatitis B vaccination among the general population due to the high efficacy of the vaccine, except in special cases and among health personnel.

As health personnel are at greater risk due to their work routine, in addition to completing the hepatitis B vaccination schedule, it is extremely important to have an anti-HBs test so that they know their immune status against the hepatitis B virus.^6,7,8^

Health personnel are recommended to undergo anti-HBs serology 1 to 2 months after the last dose of the vaccine to check whether there has been a satisfactory response to the vaccine or vaccine failure (anti-HBsAg > 10 IU/L). Health personnel who have already had contact with the virus are immune to re-infection.^2^

Persons in risk groups, such as health personnel, who have been vaccinated with three doses but have not responded with adequate levels of protective antibodies, should be revaccinated with three more doses. Those who remain unresponsive, even after two full three-dose schedules, should be considered non-responders and susceptible in the event of exposure to the hepatitis B virus.^2^

Among health personnel who reported full hepatitis B vaccination and were tested for HBs, 28.1% showed no seroconversion after vaccination. It is known that these levels decrease as time progresses.^19,20,23,24^ Nevertheless, factors such as the age at which the vaccine was received and sex have a significant influence on the immune response.^23,24^

In a study conducted in Minas Gerais, Brazil, coverage of full vaccination against hepatitis B was 52.5% and, when testing for anti-HBs, 16.4% of those who received the vaccine were not immune.^25^ The prevalence of full vaccination among health personnel in Bahia, Brazil, was 59.9%: registered nurses and physicians were more immunized; however, 13.4% of health personnel said they had not acquired protection through vaccination.^8^ It is therefore understood that testing is the only procedure for monitoring the immune response to vaccination; however, the indication of serology for health personnel is still not widely publicized in Brazil. The need for booster doses of the hepatitis B vaccine after a series of primary vaccinations is still the subject of much debate.

The limitations of the study include biases related to memory and false response. Memory bias and false response bias are due to the impossibility of checking, on the vaccination records, whether the information the health personnel have self-reported is in fact true. Therefore, there is the possibility of the health personnel not remembering the answer or choosing to provide an expected answer for it because the questioning addresses an expected positive action or behavior, such as receiving the three doses of the hepatitis B vaccine.

## CONCLUSIONS

Vaccinating adults in Brazil is a major challenge, even among health personnel. In this study, there was a great disparity in the prevalence of full hepatitis B vaccination between health care settings: 59.7% in PHC and 47.5% in medium complexity. This scenario is closely related to the PHC routine, which is responsible for vaccination in general. In this sense, in addition to the need for actions to raise awareness and encourage vaccination, in order to increase the prevalence of hepatitis B vaccination in PHC, it is also essential to introduce management and communication strategies focused on medium-complexity health personnel to complete vaccination schedules.

One third of the health personnel who reported having completed their hepatitis B vaccination did not have adequate levels of protective antibodies, and were therefore exposed to viral infection, perhaps because they were unaware of the need to check their immunity or because of the cost of the test, which is not free of charge. This reinforces the need, in addition to actions aimed at encouraging vaccination, for strategies that encourage health personnel to take the anti-HBs test and provide it free of charge or cover the cost of the test, especially for those who are more exposed to occupational risk.

Finally, vaccination should not be seen only as a health personnel self-care action, disconnected from health care and health service management actions. Therefore, strategies such as continuous monitoring of vaccination coverage, health education for health personnel, encouragement to complete the vaccination schedule in routine vaccination drives and testing for anti-HBs, targeting health personnel. Implementing technologies for communicating overdue vaccinations can favor the achievement of full hepatitis B schedules and immunity testing among health personnel.
